# A Neonatal Piglet Model for Investigating Brain and Cognitive Development in Small for Gestational Age Human Infants

**DOI:** 10.1371/journal.pone.0091951

**Published:** 2014-03-17

**Authors:** Emily C. Radlowski, Matthew S. Conrad, Stephane Lezmi, Ryan N. Dilger, Brad Sutton, Ryan Larsen, Rodney W. Johnson

**Affiliations:** 1 Department of Animal Sciences, University of Illinois, Urbana, Illinois, United States of America; 2 Division of Nutritional Sciences, University of Illinois, Urbana, Illinois, United States of America; 3 Neuroscience Program, University of Illinois, Urbana, Illinois, United States of America; 4 Department of Veterinary Pathobiology, University of Illinois, Urbana, Illinois, United States of America; 5 Department of Bioengineering, University of Illinois, Urbana, Illinois, United States of America; 6 Biomedical Imaging Center, Beckman Institute, University of Illinois, Urbana, Illinois, United States of America; Hôpital Robert Debré, France

## Abstract

The piglet was investigated as a potential model for studying brain and cognitive deficits associated with being born small for gestational age (SGA). Naturally farrowed SGA (0.7–1.0 kg BW) and average for gestational age (AGA, 1.3–1.6 kg BW) piglets were obtained on postnatal day (PD) 2, placed in individual cages, and provided a nutritionally adequate milk replacer diet (285 ml/kg/d). Beginning at PD14, performance in a spatial T-maze task was assessed. At PD28, piglets were anesthetized for magnetic resonance (MR) imaging to assess brain structure (voxel-based morphometry), connectivity (diffusion-tensor imaging) and metabolites in the hippocampus and corpus callosum (proton MR spectroscopy). Piglets born SGA showed compensatory growth such that BW of SGA and AGA piglets was similar (P>0.05), by PD15. Birth weight affected maze performance, with SGA piglets taking longer to reach criterion than AGA piglets (p<0.01). Total brain volume of SGA and AGA piglets was similar (P<0.05), but overall, SGA piglets had less gray matter than AGA piglets (p<0.01) and tended to have a smaller internal capsule (p = 0.07). Group comparisons between SGA and AGA piglets defined 9 areas (≥ 20 clusters) where SGA piglets had less white matter (p<0.01); 2 areas where SGA piglets had more white matter (p<0.01); and 3 areas where SGA piglets had more gray matter (p<0.01). The impact of being born SGA on white matter was supported by a lower (p<0.04) fractional anisotropy value for SGA piglets, suggesting reduced white matter development and connectivity. None of the metabolites measured were different between groups. Collectively, the results show that SGA piglets have spatial learning deficits and abnormal development of white matter. As learning deficits and abnormalities in white matter are common in SGA human infants, the piglet is a tractable translational model that can be used to investigate SGA-associated cognitive deficits and potential interventions.

## Introduction

Rapid growth during the last trimester and early postnatal life makes the brain particularly vulnerable to insults [1] including premature delivery [2], intrauterine growth retardation [3] and malnutrition [4]. Infants with low birth weight (LBW) are at higher risk of morbidity and mortality during their early months or years and have a tendency to develop metabolic abnormalities in the future [5]. Infants born with a LBW show a higher incidence of cognitive deficits that persist into adulthood [6], [7]. This is an enormous problem because more than 20 million infants are born each year with LBW [8]. Enhancing brain and cognitive development in LBW infants is critically important as cognitively impaired children adapt poorly to stressful events and are more vulnerable to anxiety and attention deficit disorders [9], [10]. In many parts of the world, including the U.S., cognitive impairment contributes to the cycle of poverty and disease [11], [12]. Moreover, cognitive dysfunction is a major co-morbidity in a number of neuropsychiatric diseases that manifest later in life [13]. Thus, understanding the mechanisms underlying delayed brain and cognitive development is essential to develop efficacious interventions for reversing or mitigating cognitive deficits associated with LBW.

Progress on understanding underlying factors influencing brain and cognitive development in LBW infants has been slow because studies in human infants are either impossible, due to obvious ethical considerations, or extremely difficult. Furthermore, results from rodent models commonly used to investigate neurodevelopment are difficult to translate to LBW human infants due to the substantial differences in brain development and morphology. In this regard, the domestic piglet may be an excellent model. Similar to humans, the major brain growth spurt in pigs extends from the late prenatal to the postnatal period [14]. Gross anatomical features, including gyral pattern and distribution of gray and white matter of the neonatal piglet brain are similar to that of human infants [15], [16]. Moreover, their physical size allows neuroimaging instruments designed for humans to be used with piglets. Indeed, structural magnetic resonance imaging (MRI), functional MRI, and positron emission tomography have all been conducted in pigs [17], [18], [19], [20]. Finally, due to their precocial nature, piglets can be weaned 1–2 d after birth, maintained with relative ease, and used in behavioral testing paradigms to assess learning at an early age [21], [22]. Thus, piglets represent a gyrencephalic species with brain growth similar to humans that can be used in highly controlled experiments to explore how LBW affects brain structure and function.

The natural variance that occurs in birth weights between piglets of the same litter, mostly due to decreased passage of adequate nutrition from sow to some piglets, can be used to model intrauterine growth restriction (IUGR), which is observed in approximately 24% of newborn human infants every year [23]. IUGR can be defined as impaired growth and development of mammalian embryo/fetus or its organs during pregnancy [24]. In pigs, IUGR in naturally occurring, and mostly related to placental insufficiency and multifetal pregnancy [24], [25]. In pigs, placental position and placental size can effect growth of the fetus [24] with some “runts” only being one half to one third the size of the largest littermates [26]. In humans, just like in pigs, IUGR frequently leads to small for gestational age (SGA) neonates. An infant is classified as SGA if its birth weight is in the lowest 10^th^ percentile [27]. These infants are not pre-term, but born at term (at least 37 weeks gestation, [27]) and are LBW. The incidence of SGA in developed countries is approximately 10%, while the incidence in developing countries can reach 60% [28]. Just as other LBW infants, SGA babies are at a higher risk of having learning difficulties and cognitive deficits that persist into adulthood. Importantly, SGA piglets and SGA human infants show a comparable level of maturity at birth [29]. Since artificial insemination is used in our system and time of conception is known, the effects of being SGA can be distinguished from the effects of being born preterm and LBW. In humans, determining gestational age can be difficult [30]. Therefore, the SGA piglet may be a model for human infants born SGA due to IUGR. Therefore, in the present study we hypothesized SGA piglets would have delayed postnatal brain and cognitive development compared to average for gestational age (AGA) piglets.

## Materials and Methods

### Animals, Housing, and Feeding

Littermate pairs of naturally farrowed, newborn piglets (AGA, 1.3–1.6 kg, n = 7; SGA, 0.7–1.0 kg, n = 7) were obtained from 5 separate litters from the University of Illinois swine herd. Piglets were brought to the biomedical animal facility 48 h after birth (to allow for colostrum consumption from the dam) and housed individually in cages (0.76 m L×0.58 m W×0.47 m H) designed for neonatal piglets. Each cage was positioned in a rack, with stainless steel perforated wall partitions and clear acrylic front and rear doors. In addition, each cage was fitted with flooring designed for neonatal animals (Tenderfoot/NSR, Tandem Products, Inc., Minneapolis, MN, USA). A toy (plastic Jingle Ball™, Bio-Serv, Frenchtown, NJ, USA) was provided to each piglet. Room temperature was maintained at 27°C and each cage was equipped with an electric heat pad (K&H Lectro-Kennel™ Heat Pad, K&H Manufacturing, LLC, Colorado Springs, CO, USA). When in contact with the piglet's body, the pad could reach a maximum of 40°C. Piglets were maintained on a 12-h light/dark cycle; however, during the dark cycle minimal lighting was provided.

Piglets were fed a nutritionally complete non-medicated commercial piglet milk replacer (Advance Liqui-Wean, Milk Specialties Co., Dundee, IL, USA). Milk was reconstituted fresh each morning to a final concentration of 206 g/L using tap water and supplied at a rate of 285 ml/kg BW (based on daily recorded weights). This level of feeding allowed for maintenance and growth, but prevented complete satiation to ensure that the piglets remained motivated for food rewards in the behavioral task. Water was not provided separately from that used in the milk replacer. Milk replacer was delivered from a reservoir to a stainless steel bowl (secured to the side of each cage) via a peristaltic pump (Control Company, Friendswood, TX). Using this automated delivery system (similar to that described previously [31], piglets received their daily allotted milk over 18 meals (once per hour), followed by a 6-h fasting period prior to behavioral testing where no milk was provided. All animal care and experimental procedures were in accordance with the National Research Council Guide for the Care and Use of Laboratory Animals and approved by the University of Illinois at Urbana-Champaign Institutional Animal Care and Use Committee. All efforts were made to minimize suffering.

### Cognitive Testing using a Spatial T-Maze Task

Piglet spatial learning and memory was assessed using a clear plastic plus-shaped maze (essentially a double T-maze) with extra maze cues, which has been previously described and validated [32]. The maze consisted of two start arms (north and south) and two reward arms (east and west). Start arm location was alternated throughout testing, which ensured that the piglet did not solve the task using an egocentric mechanism (i.e., turn body left or right, striatum-dependent), and instead was forced to adopt an allocentric mechanism (i.e., used extra-maze visual cues to create a spatial map of the room, hippocampus-dependent) for solving the task [33]. This task is similar to that used in rodents to assess ‘place’ and/or ‘direction’ learning [34]. Starting on post-natal day (PD) 14, piglets were tested daily between 08:00 h and 12:00 h by one trained experimenter. Piglets completed 10 trials per day (60 s per trial), for a total of 10 days. The first 6 days of testing constituted the acquisition phase, where piglets learned to locate the chocolate milk reward (3 ml of the same milk replacer used for regular feedings with the addition of Nesquik cocoa powder, supplied according to the manufacturer's directions) in a constant place in space, as well as direction (e.g., west reward arm), using the extra-maze visual cues. Chocolate milk was provided in both reward arms to balance for olfactory cues, but was only accessible in the correct reward arm. A performance criterion of 80% correct was applied, which when reached, would indicate that the piglets had successfully acquired the task. Acquisition was followed by a reversal phase of testing, where the previously incorrect arm (e.g., east), was now rewarded. A video camera was mounted from the ceiling above the arena and used to record piglet movement within the maze. Piglet movement was tracked live using commercially available software (EthoVision 3.1; Noldus Information Technology Inc., Leesburg, VA, USA).

### Magnetic Resonance Imaging

On PD 28, piglets were transported to the Biomedical Imaging Center at the Beckman Institute and anesthetized using a telazol:ketamine:xylazine (100/50/50 mg/kg/BW); Fort Dodge Animal Health]. Anesthesia was maintained by inhalation of isoflurane (98% oxygen/2% isoflurane). A MRI compatible pulse oximeter was used to monitor piglet vital signs throughout the scanning procedure. Observational records were taken every 15 minutes after an animal had been anesthetized and through the complete recovery period. After piglets had entered a sustainable plane of deep anesthesia, they were placed in the MRI machine. Piglets were restrained and remained immobilized throughout the MRI scan to prevent motion artifacts in the subsequent images.

All MRI was conducted using a Siemens MAGNETOM Trio 3 T imager and a 32-channel head coil (Siemens, Erlangen, Germany). For structural analyses (i.e., volume of discrete brain regions), anatomic images were acquired using three repetitions of a 3D T1-weighted magnetization-prepared rapid gradient-echo sequence with the following parameters: repetition time  = 1,900 ms; echo time  = 2.49 ms; inversion time  = 900 ms, flip angle  = 9°, matrix  = 256×256, 224 slices with thickness  = 0.7 mm. The final voxel size was 0.7 mm isotropic across the entire head from the tip of the snout to the cervical/thoracic spinal cord junction as described previously [35].

Analysis of neuronal connectivity was conducted in multiple brain regions using a DTI sequence to visualize fiber tracts non-invasively. A b-value of 1000 sec/mm^2^ and 30 directions was used, and data were processed using a publically available software toolset, the FMRIB Software Library (FSL). Finally, quantitative data on brain metabolite concentrations was collected using MR-Spectroscopy. Due to motion artifacts, all MRI based analysis has only 5 piglets per group (AGA, n = 5; SGA, n = 5).

### Voxel-Based Morphometry and Brain Region Volume Estimation

After conversion from the DICOM format to the NIfTI format, the three individual MPRAGE scans were averaged. Next, using FSLView of the FSL package, a mask was manually drawn over the brain and then used for brain extraction (Analysis Group, FMRIB, Oxford, UK) [36] The remainder of the voxel-based morphometry analysis was conducted in SPM8 (Wellcome Department of Clinical Neurology, London; http://www.fil.ion.cul.ac.uk). The extracted brains were translated into the standard brain space and then aligned to a piglet brain atlas via a 12 parameter affine transformation. Using prior probability maps specific for the piglet, brains were segmented into gray matter, white matter, and cerebrospinal fluid maps using the “Segment” function of SPM [37]. This function uses a combination of prior probabilities, mixture of Gaussians, and registration terms for tissue classification [37]. Changes to the default settings include substituting the human priors with the piglet priors and no affine regularization.

The Diffeomorphic Anatomical Registration using Exponentiated Lie Algebra (DARTEL) package was used to create study specific templates [38]. DARTEL is considered to be a step forward in segmentation analysis. The gray and white matter segmented data sets were simultaneously used to create a series of six templates. Changes from default include a bounding box of −30.1 to 30.1, −35 to 44.8, −28 to 31.5; and a voxel size of 0.7 mm^3^. A linear bend energy was used as the cost function. After a nonlinear transformation in the DARTEL procedure, flow fields were generated which were converted to warp fields. Warp fields represent regional expansion or shrinkage of the subject brain to atlas space. These warp fields were applied to the subject gray and white matter and these modulated data were smoothed with a 4 mm full-width half maximum (FWHM). The modulated gray and white matter were then subjected to voxel-based morphometry analysis using the statistical non-parametric methods (SnPM) toolbox described below. Overlay images were created using the VBM8 toolbox (http://dbm.neuro.uni-jena.de/vbm.html).

Larger region of interest analysis was also conducted. Using the warp fields generated during the DARTEL process, an inverse warp was created in SPM for each subject representing the expansion/shrinkage from the atlas to subject space. These inverse warp fields were then applied to the regions of interest (ROI) found in the atlas package. The volumes were then measured for each ROI for each subject. The resultant images were smoothed with a 4 mm full-width half maximum (FWHM).

### Diffusion Tensor Imaging

The diffusion tensor imaging (DTI) measured global white matter, global gray matter, and corpus callosum within the brains of SGA and AGA piglets. The corpus callosum was chosen because of the high concentration of white matter tracts within the area. The DTI acquisition used a diffusion-weighted echo-planar-imaging (DW-EPI) sequence with the following parameters: repetition time  = 5000 ms; echo time  = 91 ms; averages  = 3; diffusion weightings  = 2; b-value of 1000 s/mm^2^ across 30 directions; two images with a b-value of 0 s/mm^2^. Forty slices with a 2.0 mm thickness were collected with a matrix size of 100×100 for a final voxel size of 2.0 mm isotropic.

**Figure 1:**
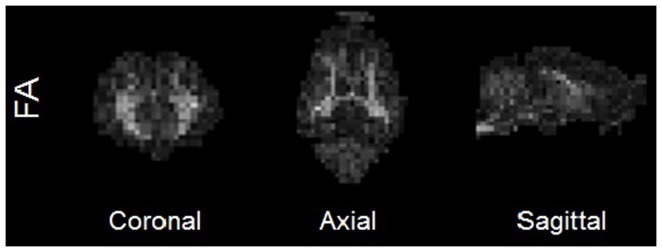
FA maps of a representative pig in coronal, axial, and sagittal sections.

The DW-EPI images were then analyzed using the diffusion toolbox in the FSL software package (http://fsl.fmrib.ox.ac.uk/fsl/fsl-4.1.9/fdt/) to create fractional anisotropy (FA) images. Masks of the corpus callosum and white matter from the piglet brain atlas were nonlinearly transformed to the subject's MPRAGE space and then linearly transformed into the DTI space using FSL. The corpus callosum region of interest had a threshold of 0.15 applied to compensate for expansion caused by interpolation. The white matter ROI had a threshold of 0.5 applied and was dilated twice. Averages were taken in the regions of interest with a FA>0.2 mask to select only white matter tracts. Averages were taken over the whole brain with a FA>0.2 mask to assess global FA values. The average FA values in the white matter tracts of the left and right half of the brain were also computed. An example FA map can be found in **Figure 1**.

### Magnetic Resonance Spectroscopy

Magnetic resonance spectroscopy (MRS) was performed using a spin echo chemical shift sequence with the following parameters: repetition time  = 3000 ms; echo time  = 30 ms; one average; FOV = 72 mm with an 16×16 matrix; 10 mm thickness. The final voxel size was 4.5×4.5×10 mm. Eight presaturation bands were placed around the volume of interest (hippocampus and corpus callosum). Automatic and/or higher-order shims were conducted before each spectroscopic scan to ensure a full width-at-half-maximum (FWHM) of less 18 Hz. Both water-suppressed and non-water suppressed data were collected.

**Figure 2:**
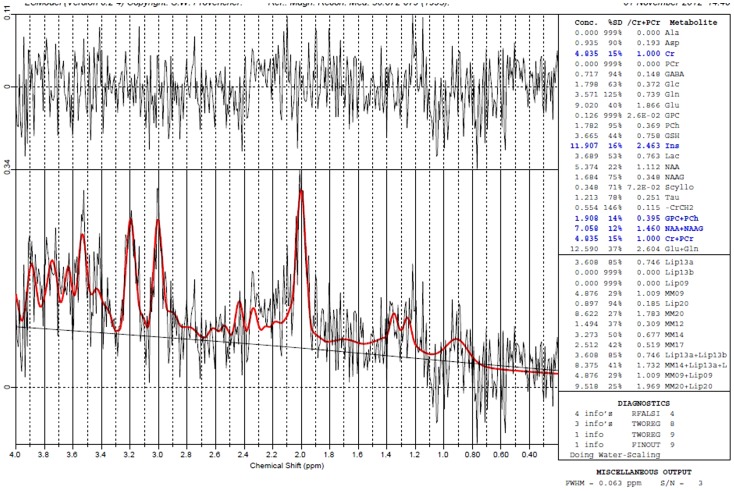
Representative example of piglet Magnetic Resonance Spectroscopy Spectra.

All MRS data files were subsequently analyzed by LC Model 6.3 fitting program. The LC Model method analyzes in vivo spectra as a linear combination of model in vitro spectra from individual metabolite solution [39]. Absolute levels, in institutional units, were obtained by using an eddy current correction procedure and water scaling. Water-suppressed time domain data were analyzed between 0.2 and 4.0 ppm without further T_1_ or T_2_ correction. There was no post-suppression of water while using the LC Model. The basis-set of metabolite phantom spectra was used as prior knowledge; the impact of lack of post-suppression was minimal. Absolute concentrations were calculated for each metabolite. Cramer-Rao lower bounds (S.D.%) were calculated by the LC Model to evaluate the quantitative result of absolute levels. Only those levels that were less than 20% were considered reliable and were included for further statistical analysis. The spectral data from the voxels in the left and right hippocampus were averaged to create an averaged hippocampus spectrum. A single voxel in the corpus callosum was selected for each subject. An example of the spectra can be seen in **Figure 2**.

Creatine and phosphocreatine were used as internal standards. NAA tends to be the most prominent signal in MRS of the human brain and is located in pyramidal neurons, dendrites, axons, and oligodendrocytes [40], [41] within the CNS and is used to signify viable brain cells and denote neural density [42], [43]. Choline is used as a measure of myelin viability; when active myelin is damaged and degraded, there is a release of phospholipid and a spike in the choline peak on the spectrum [44]. Myo-inositol is a marker for astrocytes [41] as well as a glial cell marker and is used in several physiological functions [45]; while glycerol 3-phosphocholine is important in the synthesis of acetylcholine and is used in brain metabolism [46]. Lactate can be used as a measure of ischemia or inflammation in the brain [47].

### Brain Weight

After MRI was complete, animals were re-administered 0.5 mL telazol:ketamine:xylazine solution to maintain anesthesia and then euthanized by intracardiac injection of sodium pentobarbital (Fatal Plus, 72 mg/kg body weight). All efforts were made to minimize suffering. After euthanasia, whole brain tissue, excluding optic nerves, was collected and weighed.

### Statistical Methods

#### Weight gain, brain weight, and behavior

Data analysis was conducted using the MIXED procedure of the SAS software (SAS Institute). Weight gain and cognitive task data were analyzed as a 2-way (treatment*day) repeated measures ANOVA. Analysis of brain weight data used the MIXED procedure of the SAS software. Brian weight was analyzed as a 1-way (treatment) ANOVA. For all analyses, measures that were not significant (sex, rep, litter) were removed from the model. Post-hoc paired contrasts were used to further examine treatment effects. Significance was accepted at p<0.05. Data are presented as average ±SEM.

#### Voxel-Based Morphometry

The statistical non-parametric methods toolbox was used for statistical analysis (http://go.warwick.ac.uk/tenichols/snpm) [48]. This package uses non-parametric permutation and randomization tests which are beneficial when there are a small number of individuals per treatment. A two-sample t-test design was used to compare AGA versus SGA. No covariates were used. An ANCOVA was used for global normalization. Pseudo-T statistic maps were generated showing areas where there was a reduction in gray or white matter in the SGA piglets using an uncorrected p<0.01 to allow more voxels to be detected. A threshold of at least 20 edge-connected voxels (clusters) was used.

#### Brain Region Volume Estimation

The brain region volume estimates were subjected to the T-TEST procedure of the SAS software (SAS Institute). A two-sample two-sided t-test design was used to compare the brain region volume estimates between AGA versus SGA. Significance was accepted at p<0.05.

#### DTI and MR-Spectroscopy

Data analysis was conducted using the T-TEST procedure of the SAS software (SAS Institute). A two-sample one-sided t-test design was used to compare AGA versus SGA for the DTI data and a two-sample two-sided t-test was used for MR-spectroscopy. No covariates were used. Significance was accepted at p<0.05. Data are presented as average ±SEM.

## Results

### Brain and Body Weights

**Figure 3:**
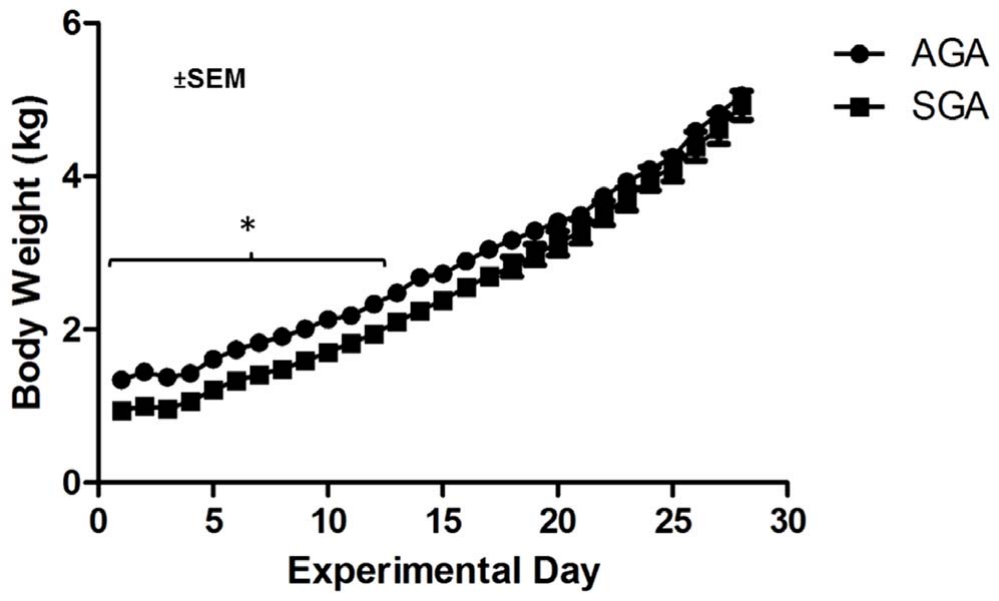
Body weight of AGA and SGA piglets during the study. Body weight of AGA and SGA piglets was different (*p<0.05) until PD14 but not thereafter. Data are shown as the mean ± SEM (n = 7).

AGA piglets were born with an average body weight of 1.34±0.013 kg and SGA piglets were born with an average body weight of 0.94±0.19 kg (p<0.05). The average body weights of AGA and SGA piglets were different (p<0.05) from 0 to 14 days of age but not thereafter (**Figure 3**). As AGA and SGA piglets were fed 285 ml/kg body weight, these data indicate SGA piglets experienced compensatory “catch-up” growth. Moreover, AGA and SGA piglets had similar body weights when cognitive testing commenced. At PD 28, average brain weights for SGA and AGA piglets (33.9 g and 34.9, respectively) were not different (p>0.10).

### Cognitive Testing

**Figure 4:**
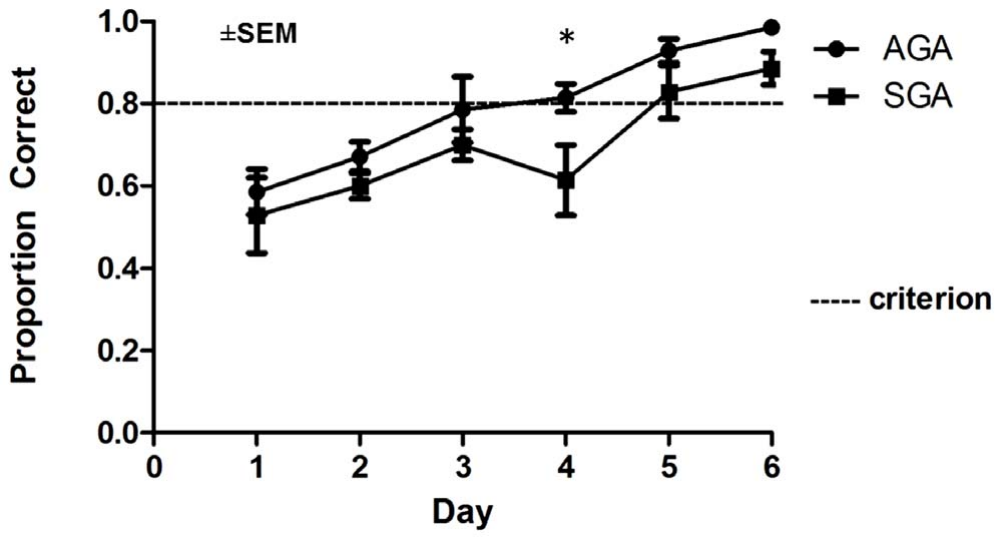
Performance of AGA and SGA piglets during the acquisition phase of a spatial T-maze task. Performance of AGA and SGA piglets improved over time (p<0.001) but AGA piglets reached criterion 2 d sooner than SGA piglets. The asterisk indicates performance of SGA piglets was lower on d 4 compared to AGA piglets (p = 0.01). Data are shown as the mean ± SEM (n = 7).

Birth weight altered piglet performance in the spatial T-maze task (p<0.05), where SGA piglets performed more poorly in the acquisition phase than AGA piglets, as indicated by fewer correct choices (**Figure 4**). Performance of both SGA and AGA piglets improved over time (p<.0001) but the treatment*day interaction was not significant (p>0.05). However, paired contrasts between treatments on d 1-6 of acquisition revealed a significant learning deficit in SGA piglets. Whereas AGA piglets reached criterion (80% correct) by d3 of acquisition, SGA piglets required an additional 2 days of training to reach criterion. The additional training needed for SGA piglets to reach criterion was verified in two independent trials.

During the reversal phase, there were no significant differences (p>0.05) in learning ability between the SGA and AGA piglets (data not shown). All piglets improved over time (p<0.001) but neither group reached criterion. There were no treatment*day interactions and paired contrasts did not show any significant differences between treatments.

### Magnetic Resonance Imaging

#### Voxel-based morphometry

The SGA group showed a significant reduction (p<0.01) in global gray matter volume when compared to AGA piglets. There was also a trend (p = 0.07) for SGA piglets to have a smaller internal capsule volume when compared to AGA piglets. These comparisons, as well as the rest of the estimated volumes for each brain region, are shown in **Table 1**.

**Table 1:** Estimated volumes by brain region in SGA versus AGA piglets.

Region	AGA mm^3^ (SE)	SGA mm^3^ (SE)	P-value
Whole Brain	69961 (1650.7)	68218 (1315.7)	0.433
White Matter	11018 (805.1)	10446 (1519.6)	0.748
**Gray Matter**	**17205.2 (257.1)**	**15815.8 (248.8)**	**0.005**
Caudate	573.4 (32.04)	559.4 (33.68)	0.771
Cerebellum	4934.4 (103.7)	4883 (172.3)	0.805
Cerebral Aqueduct	123.4 (2.82)	134.2 (4.3)	0.09
Corpus Callosum	998.4 (18.32)	995.6 (14.09)	0.907
Fourth Ventricle	141.6 (7.56)	154.4 (7.01)	0.25
Hypothalamus	523.8 (16.83)	559.4 (11.03)	0.115
**Internal Capsule**	**4986.4 (120.4)**	**4696.6 (73.67)**	**0.074**
Lateral Ventricle	1075.6 (45.83)	1076.4 (25.60)	0.998
Left Cortex	17325.8 (371.7)	18016 (614.9)	0.365
Left Hippocampus	603.2 (22.59)	594.4 (19.53)	0.776
Medulla	2528 (43.52)	2517.4 (34.9)	0.854
Midbrain	3069 (71.96)	3043.8 (39.35)	0.767
Olfactory Bulb	2898 (76.83)	3110.2 (99.08)	0.129
Pons	856 (29.62)	874.8 (12.67)	0.576
Putamen	1226.8 (35.72)	1161.8 (23.87)	0.169
Right Cortex	16292.4 (326.6)	16890.6 (559.2)	0.383
Right Hippocampus	655.2 (22.36)	664.8 (16.38)	0.738
Thalamus	3113 (67.25)	3055.4 (52.74)	0.519
Third Ventricle	207.8 (12.84)	206.4 (6.97)	0.926

Those regions that are bolded were found to be significant. Values are represented as Mean ± SE. Statistical significance is set at a p<0.05.

**Figure 5:**
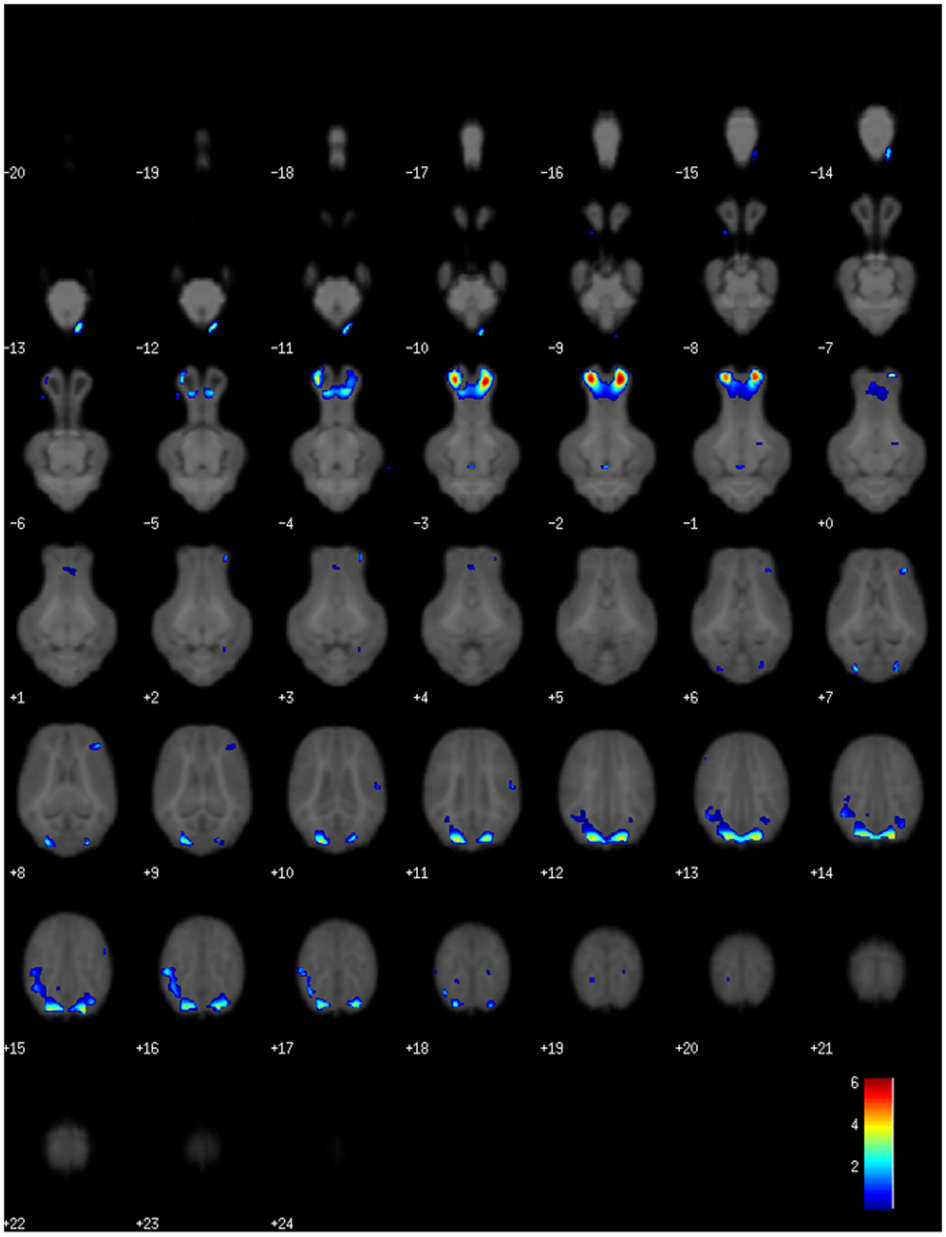
Voxel-based morphometry analysis using DARTEL showed increased gray matter volumes in the olfactory bulb and cortex of SGA piglets. The color bar indicates the pseudo-t statistic indicating level of significance.

**Figure 6:**
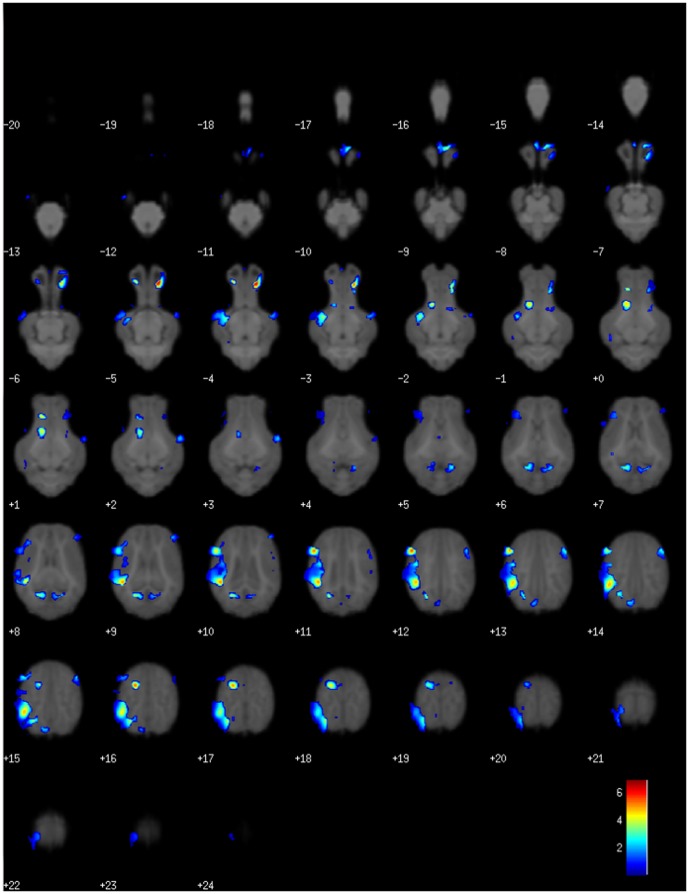
Voxel-based morphometry analysis using DARTEL defined 9 areas where SGA piglets had less white matter than AGA piglets. These areas were in the olfactory bulb, left and right cortex, and internal capsule. The color bar indicates the pseudo-t statistic indicating level of significance.

In the VBM-DARTEL analyses (**Table 2**), despite SGA having a global reduction in gray matter, the group comparison (SGA>AGA) showed SGA had greater gray matter volumes in several brain regions including, the olfactory bulb, as well as the left and right cortices (**Figure 5**). An adult brain atlas was used to estimate which areas within the left and right cortices our coordinates mapped to. Within both cortices, the coordinates mapped to the primary and associative visual cortex. In white matter, group comparisons (SGA>AGA) showed that SGA piglets had greater white matter volumes in the cerebellum and mid-brain. However, the most notable difference between treatment groups was that SGA piglets had 9 brain areas with decreased white matter volumes when compared to AGA piglets (group comparison SGA<AGA). These areas mapped to the olfactory bulb, left cortex, right cortex, and internal capsule (**Figure 6**). Within the cortices, the coordinates generated from our analysis mapped to the primary and secondary visual cortex and primary somatosensory cortex on the left side, and the primary visual cortex and amygdala on the right side. This voxel wise comparison (SGA<AGA) showed no significant differences in gray matter within individual brain regions.

**Table 2:** Voxel-based morphometry analysis of Gray Matter and White Matter differences in the SGA and AGA piglet brains. Non-parametric permutation tests were used with a threshold of p<0.01 uncorrected and a minimum cluster size of 20 voxels.

				Cluster	Cluster level	Local	Maxima	Coordinates*	
	Comparison	Anatomic Region	**AnatomicSub-Region	(voxels)	(p-value)	x	y	z	*Pseudo-t*
	**SGA<AGA**	No significant findings							
		Olfactory Bulb		1618	0.004	6	33	-2	6.18
Gray Matter		Olfactory Bulb			0.0079	-7	34	-2	5.85
	**SGA>AGA**	Left Cortex	PVC	1432	0.0079	-6	-17	13	4.22
		Right Cortex	PVC		0.0079	8	-16	13	4.22
		Right Cortex	AVC	20	0.0079	10	-18	8	3.58
		Olfactory Bulb		844	0.0079	7	28	-4	6.89
		Left Cortex	SVC	5414	0.0079	-15	-1	10	5.12
		Left Cortex	PVC		0.0079	-8	-11	10	4.21
		Left Cortex	PSC	295	0.0079	-8	14	16	5.34
	**SGA<AGA**	Left Cortex	PSC	49	0.0079	-4	24	1	4.96
White Matter		Olfactory Bulb		50	0.0079	-6	29	-4	4.91
		Internal Capsule		264	0.0079	-5	15	-1	4.79
		Left Cortex	Amygdala	477	0.0079	-12	7	-3	3.56
		Right Cortex	PVC	206	0.0079	8	-9	8	3.22
		Left Cortex	PVC	123	0.0079	-3	-13	15	2.26
	**SGA>AGA**	Cerebellum		87	0.004	-8	-14	-6	3.24
		Midbrain		62	0.0079	3	-1	1	2.25

*X increases from left (-) to right (+), y increases from posterior (-) to anterior (+), and z increases from inferior (-) to superior (+).

** Based on estimates from adult brain atlas. PVC- primary visual cortex, AVC-associated visual cortex, SVC- secondary visual cortex, PSC- primary somatosensory cortex.

#### Diffusion tensor imaging and magnetic resonance spectroscopy

**Figure 7:**
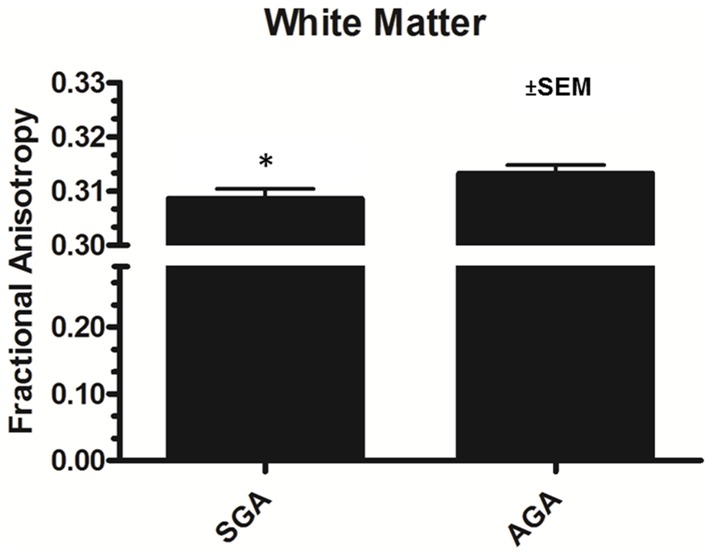
Fractional anisotropy (FA) values for AGA and SGA piglets. The FA value determined from Diffusion Tensor Imaging was lower in SGA piglets compared to AGA piglets (*p = 0.04). Data are shown as the mean ± SEM (n = 5).

Differences in global gray and white matter and corpus callosum white matter tract development were evaluated between SGA and AGA piglet groups. SGA piglets showed a significant (p = 0.04) decrease in FA within global white matter compared to AGA piglets (**Figure 7**), indicating a reduction in neural integrity or differences in brain microstructure. There were no significant differences within the corpus callosum or global gray matter. Mean diffusivity (MD) values were analyzed and showed no significant differences between groups in global white or gray matter, or corpus callosum. Postnatal changes in MD are not as apparent in the neonatal piglet as they are in humans, which may be due prenatal changes in brain water content [49]. Absolute metabolite concentrations of creatine, choline, N-acetylaspartate, myo-inositol, glycerol 3-phosphocholine, and lactate were measured in the corpus callosum and hippocampus of SGA and AGA piglets. If the standard deviation percentage exceeded 20%, data were excluded from the analysis. There were no significant differences for any of the metabolites measured (**Table 3**).

**Table 3:** MRS in hippocampus and corpus callosum of AGA and SGA piglets.

		AGA	SGA
		(SEM)	(SEM)
Region	Metabolite*	(n = 5)	(n = 5)
**Hippocampal**			
	NAA	5.20 (0.27)	5.58 (0.39)
			
	GPC+PCh	1.76 (0.16)	1.79 (0.04)
			
	Cr+PCr	4.44 (0.40)	4.28 (0.18)
			
**Corpus Callosum**		(n = 5)	(n = 3)
	NAA	5.02 (0.24)	6.82 (0.58)
			
	GPC+PCh	1.69 (0.12)	1.63 (0.30)
			
	Cr+PCr	4.21 (0.42)	4.28 (0.47)

There were no significant differences between groups. Myo-inositol and lactate did not meet criteria for analysis (%S.D.<20) and were not included.

*NAA, N-acetylaspartate; Cr, creatine; PCr, Phosphocreatine; GPC, glycerol 3-phosphocholine; PCh, phosphocholine.

## Discussion

Infants born SGA have an increased risk of cognitive deficits and learning disabilities that may persist through adulthood [6], [7]. Therefore, it is important to develop interventions to mitigate cognitive deficits associated with being born SGA. Intervention studies with SGA human infants, however, are extremely difficult due to obvious ethical considerations. Therefore, in the present study we investigated the neonatal piglet as a potential model for studying brain and cognitive deficits associated with being born SGA. The important results show that SGA piglets have deficits in spatial learning and memory as well as decreased development of white matter, both being consistent with earlier reports in humans born SGA [50], [51], [52]. Therefore, the neonatal piglet is a highly tractable translational animal model that can be used to investigate neural mechanisms responsible for SGA-associated cognitive deficits and potential interventions.

In the present study, piglet learning and memory ability was tested using a spatial T-maze task. This task involves the hippocampus because piglets must use an allocentric mechanism (i.e., distal visual cues) instead of a striatum-dependent egocentric mechanism (i.e., turning body left or right) [33]. Further, in a previous study piglet performance in this task was inhibited by administration of scopolamine, an anticholinergic drug that acts on the hippocampus and other related structures [32]. In human adults performing a similar spatial task, functional MRI shows activation of the hippocampus [53]. Thus, it is reasonable to suggest that results from the spatial T-maze task with piglets can be relevant to understanding spatial learning and memory in humans. This is important because spatial information provides important contextual cues for retrieving other memories and has been suggested to be a key component of episodic memory [54]. Episodic memory, while once thought to be used just for daily tasks [55] (e.g., “Where did I leave my keys?”) has been shown to be important for reading comprehension as it supports the ability to integrate ideas from the text during retrieval [56]. In addition, measures of episodic memory are part of the standardized assessments of intellectual ability [57].

Herein, SGA piglets required several additional days to reach criterion in the spatial T-maze task when compared with AGA piglets, suggesting a learning deficit. Prior to d 5 of acquisition training, SGA piglets were more hesitant to make a choice and when doing so, made more errors. We did not see any differences during the reversal phase. We believe this portion of the task was confounded by “stuck-in-set” perseveration [58] within the AGA group in which these piglets inappropriately maintained a previous framework after being introduced to a new task. Human infants also demonstrate perseveration in tasks that involve cues and object- reward tests [59]. However, our acquisition data is consistent with another study that assessed learning in older pigs that were born SGA [60]. The SGA piglets in that study showed delayed acquisition in the reversal phase of a spatial hole-board discrimination task. Importantly, a lag in learning has been demonstrated in human SGA children as well. For example, several studies have demonstrated that children born SGA perform more poorly in school [7], are more likely to be enrolled in special education classes [6] and have a lower IQ [61] compared to other children that were born AGA. These deficits are seen to persist into adulthood as well, with adults who were born SGA having higher rates of not finishing school [62] and choosing unskilled labor jobs [6] or being unemployed [62]. Although the underlying mechanism(s) responsible for deficits in learning is unclear, it is hypothesized that altered development of white matter tracts in the SGA infants is involved [63].

White matter in the brain consists of oligodendrocytes and myelinated axons. It is composed mostly of lipid and myelin, which together act as an insulator to facilitate efficient transmission of signals within and between brain regions [64]. White matter tract development begins in the prenatal period and continues through adulthood [65] but is most intense in the perinatal period [66]. Insults during the prenatal period have an impact on fetal development and can interfere with proper white matter development [66]. Conduction velocity of neural signals and functional maturation of the brain is dependent on proper myelination, which requires adequate substrate for myelin formation [66]. In the model of IUGR, substrate may be lacking due to inadequate passage of nutrients from the mother to the fetus. Alterations or decreases in white matter tract development are commonly associated with reduced IQ [67], [68] and memory impairment [69]. A recent MRI study of human SGA infants found lower FA values for white matter compared with AGA controls right after birth [51], [52], suggesting antenatal growth impacts white matter maturation. In the present study, voxel-based morphometry revealed numerous brain regions with less white matter in SGA piglets compared with AGA piglets including the amygdala. This brain region has been shown to be involved in attention [70] as well as cue-, place- and object-reward associations [70], [71], [72], [73] and may be involved in some of the differences we see with the differences in learning ability in the SGA group. Moreover, diffusion tensor imaging, which is commonly used in humans to reveal white matter disorders and to construct models of brain connectivity, suggested decreased FA values for white matter in SGA piglets compared with AGA piglets. We were not able to gain FA values from discreet brain regions due to lack of power related to losing 2 piglets per group to motion artifacts. However, the MRI and spatial learning data suggest treatments that facilitate white matter development may improve cognitive development in SGA piglets. It should be noted that the effect of an enriched environment on brain structure was not tested in this study. It is possible that enrichment provided to SGA human infants facilitates development. MR-spectroscopy is used as a non-invasive method to quantify metabolite concentrations in the brain [74]. It is useful as a clinical tool and can be used to test for different pathological conditions [47]. In the present study, we examined the concentrations of creatine, NAA, choline, myo-inositol, glycerol 3-phosphocholine, and lactate within the hippocampus and corpus callosum. Unfortunately, MRS did not reveal any significant differences in metabolites in the hippocampus or corpus callosum between SGA and AGA piglets. In retrospect this may not be surprising because voxel-based morphometry also did not reveal differences in these brain areas between SGA and AGA piglets. It should be noted that Roelants-van Rijn [45] found similar brain metabolite concentrations for SGA and AGA preterm human infants when MR-spectroscopy was performed at a single time point as was done here.

While we believe this model can be useful for investigating new therapies to mitigate the consequences of being born SGA, it is important to note some limitations of the present study. In particular, the inability to identify differences between groups in metabolites within the hippocampus and corpus callosum may have been due to the low number of subjects. Data from a number of piglets were not included in the final analysis because the metabolite concentrations detected were below the sensitivity threshold. Increasing the number of subjects and performing additional analysis of tissue collected postmortem (e.g., liquid chromatography- mass spectroscopy) would be useful.

Finally, it is noteworthy that SGA piglets demonstrated compensatory ‘catch up’ growth such that by PD15 and throughout behavioral testing, the average body weight of SGA and AGA piglets was similar. Catch up growth is common in human SGA infants and is associated with decreased mortality within the first few months after birth [75], increased stress resilience, and higher intellect compared to SGA infants that do not have catch up growth [50]. Because the present study did not include a cohort of SGA piglets that remained “small,” we cannot know if the accelerated growth improved performance in the spatial T-maze task, only that the catch up growth was not sufficient to mask the effects of being born SGA. In any case, this is an interesting and important feature of the model because catch up growth in SGA human infants also dramatically increases the risk for obesity, type 2 diabetes, hypertension, and cardiovascular disease [5], [76].

In conclusion, the present study found that SGA piglets have deficits in spatial learning and memory as well as decreased development of white matter. As learning deficits and abnormalities in white matter development are common in SGA human infants, the neonatal piglet is a highly tractable translational animal model that can be used to investigate neural mechanisms responsible for SGA-associated cognitive deficits and potential interventions.
